# Resistencia flexural de cementos resinosos: evaluación *in vitro*

**DOI:** 10.21142/2523-2754-1301-2025-228

**Published:** 2025-03-03

**Authors:** Socorro Noa-Yarasca, Ana López-Flores

**Affiliations:** 1 Division de Rehabilitacion Oral, Facultad de Ciencias de la Salud, Carrera de Estomatologia, Departamento de Posgrado Universidad Cientifica del Sur. Lima, Peru. 100005654@cientifica.edu.pe dra.lopz@gmail.com Universidad Científica del Sur Division de Rehabilitacion Oral Facultad de Ciencias de la Salud, Carrera de Estomatologia, Departamento de Posgrado Universidad Cientifica del Sur Lima Peru 100005654@cientifica.edu.pe dra.lopz@gmail.com

**Keywords:** cementos autoadhesivos, resistencia flexural, propiedades mecánicas, self-adhesive cements, flexural strength, mechanical properties

## Abstract

**Objetivo:**

Evaluar la resistencia flexural de tres cementos resinosos dentales.

**Materiales y métodos:**

Se confeccionaron sesenta barras con medidas de 25 x 2 x 2 mm de los cementos RelyX-U200, Relyx^TM^ Universal y Duo-Link Universal. Estas barras se dividieron en 3 grupos según la marca (n = 20) y se mantuvieron en agua destilada por 24 horas a 37 ºC. Se llevó a cabo la prueba de flexión de 3 puntos, de acuerdo con la norma ISO 4049, en una máquina de ensayo universal a una velocidad de 0,5 mm/min hasta la fractura. El análisis estadístico se realizó con el paquete estadístico Python, mediante la prueba Anova de un factor y el test de comparaciones múltiples de Tukey, con un nivel de confianza del 95%.

**Resultados:**

El cemento Duo-Link Universal obtuvo el mayor promedio con respecto a la resistencia flexural (104.7±7.8 MPa). Se encontró diferencias estadísticamente significativas entre los tres grupos (p < 0,001). Estas diferencias se presentaron entre los cementos Relyx^TM^ Universal - Duo-Link Universal (p < 0,001) y Duo-Link Universal - RelyX U200 (p < 0,001), mientras que entre el cemento Relyx^TM^ Universal y RelyX U200 no se encontraron diferencias estadísticamente significativas.

**Conclusión:**

El cemento Duo-Link Universal presenta mayor resistencia flexural en comparación con los cementos RelyX-U200 y RelyX^TM^ Universal. Los resultados indican que Duo-Link Universal es ideal para restauraciones que requieren alta resistencia flexural, pues ofrece mayor durabilidad clínica, especialmente en zonas de mayor carga funcional y restauraciones extensas, como en prótesis indirectas o postes.

## Abstract (en)

**Objective:**

To evaluate the flexural strength of three dental resin cements.

**Materials and methods:**

Sixty bars measuring 25x2x2 mm were made of RelyX-U200, Relyx^TM^ Universal and Duo-Link Universal cements. These bars were divided into 3 groups according to brand (n=20) and were kept in distilled water for 24 hours at 37ºC. The 3-point bending test was carried out, according to ISO 4049, in a universal testing machine at a speed of 0.5 mm/min until fracture. Statistical analysis was performed with the Python statistical package, using the one-way Anova test and Tukey's multiple comparisons test with a confidence level of 95%.

**Results:**

Duo-Link Universal cement obtained the highest average with respect to flexural strength (104.7±7.8 MPa). Statistically significant differences were found between the three groups (p< 0.001). These differences were present between Relyx^TM^ Universal - Duo-Link Universal (p<.001) and Duo-Link Universal - RelyX U200 (p< .001) cements, while no statistically significant differences were found between Relyx^TM^ Universal and RelyX U200 cements.

**Conclusion:**

Duo-Link Universal cement presents greater flexural strength compared to RelyX-U200 and Relyx^TM^ Universal cements. The results indicate that Duo-Link Universal is ideal for restorations requiring high flexural strength, offering greater clinical durability, especially in areas of greater functional load and extensive restorations such as indirect prostheses or posts.

## INTRODUCCIÓN

La resistencia flexural es una propiedad biomecánica importante que proporciona información sobre cómo un material se deforma y falla bajo una carga significativa. En el caso de los cementos dentales, esta característica es vital para evaluar su eficacia, pues asegura la durabilidad y estabilidad de las restauraciones protésicas ^1^. También es considerada una variable fundamental para determinar la capacidad de un material y soportar fuerzas de flexión sin fracturarse ^2^. 

La resistencia flexural de los materiales dentales está vinculada con otras propiedades mecánicas como el módulo de elasticidad y la resistencia a la compresión, entre otras. Esta resistencia se mide mediante la prueba de flexión de tres puntos, conforme la norma ISO 4049 y equipos especializados. Dicho método proporciona información esencial sobre la capacidad de los materiales dentales para soportar las fuerzas masticatorias ^1^^,^^2^. Estas fallas ocurren cuando las restauraciones protésicas se desprenden por fuerzas intraorales que debilitan la unión con el cemento ^3^. Por lo tanto, utilizar cementos con buenas propiedades mecánicas es esencial para asegurar la durabilidad y estabilidad de las restauraciones protésicas ^4^. En los últimos años, los cementos resinosos se utilizan con mayor frecuencia como material de elección para cementaciones ^5^; no obstante, son sensibles a la técnica de aplicación, ya que la contaminación con la saliva, sangre y otros fluidos puede comprometer el sellado marginal, y generar fallas en la adhesión ^6^. 

Los múltiples procedimientos: ácido, primer/adhesivo pueden complicar el éxito de la cementación ^7^^,^^8^. Los cementos autoadhesivos han experimentado mejoras significativas en sus propiedades biomecánicas, estéticas y adhesivas. Además, han simplificado el proceso de cementación al reducir el número de pasos previos, lo que disminuye la sensibilidad a la técnica y reduce el tiempo clínico necesario para el procedimiento ^9^^-^^12^. En el área de los cementos dentales, estos autoadhesivos se posicionan como una de las más novedosas propuestas. Al ser muy versátiles, ofrecen la posibilidad de ser empleados tanto de forma autoadhesiva como en conjunto con su sistema adhesivo correspondiente. De esta manera, se adaptan a las necesidades específicas de cada caso clínico y a las preferencias del odontólogo ^13^. Cuando se utiliza un cemento con su sistema adhesivo, se obtiene una mayor fuerza de unión tanto al esmalte como a la dentina ^14^. 

A diferencia de los autoadhesivos, la unión lograda con cementos de múltiples pasos es sensible a los cambios clínicos y requiere el cumplimiento de ciertos requisitos previos ^15^. El panorama actual de los biomateriales dentales se caracteriza por una amplia gama de cementos resinosos especialmente diseñados para fijaciones dentales. Sin embargo, para que los profesionales puedan aprovechar al máximo estos materiales, es necesario realizar evaluaciones detalladas que permitan obtener información completa sobre sus características. En este sentido, la investigación sobre la resistencia flexural de estos cementos resinosos resulta fundamental y necesario en la toma de decisiones del profesional.

Investigaciones previas han relacionado la resistencia flexural de los cementos resinosos a diferentes factores como la fotoactivación ^9^, la composición, las propiedades biomecánicas (módulo de elasticidad, microdureza, microtensión) ^16^, entre otros. Estudios recientes indican que los cementos autoadhesivos de polimerización dual muestran una resistencia flexural superior a los cementos convencionales y que diferentes condiciones de fotoactivación afectan positivamente sus propiedades mecánicas ^9^^,^^17^^-^^20^. Asimismo, encontraron que la resistencia flexural del cemento autoadhesivo RelyX U200, modificado con TiO2-nt, varía según la fotoactivación. Los nanotubos de TiO2 mejoraron la resistencia en la autopolimerización, pero no en la polimerización dual. Además, una adición del 0,6% de nanotubos redujo la resistencia flexural ^20^. 

Estudios previos hechos por Camargo *et al*. ^21^ también han demostrado que los cementos resinosos con polimerización dual y asociados a hidroxiapatita (HAp) logran una destacada resistencia flexural que supera la de cementos de polimerización dual sin HAp. Estos resultados subrayan la relevancia de la HAp y el tipo de fotoactivación en las propiedades mecánicas de los cementos resinosos, lo que ofrece valiosas perspectivas para optimizar su desempeño clínico. La resistencia flexural también fue vinculada a la temperatura de almacenamiento en los cementos resinosos. Estudios realizados por Gietzak *et al*. ^22^ evaluaron cómo varían ciertos indicadores de resistencia de cementos resinosos de polimerización dual al ser almacenados a diferentes temperaturas. Los resultados indican que la resistencia flexural a temperaturas de 25 °C muestra valores inferiores en resistencia flexural, y que a temperaturas de 35 °C se registran valores superiores. Además, se observó que el cemento con un contenido de relleno del 70% en peso presentó valores más altos en resistencia flexural y microdureza.

La resistencia flexural también fue relacionada a la composición de los cementos resinosos. Rodrigues *et al*. ^23^ han señalado que la resistencia flexural exhibe variaciones según la composición específica del cemento. En este contexto, se evidenció que los cementos resinosos exhibieron mayor resistencia, lo que podría estar asociado con las diferencias en la composición química y las propiedades de los materiales de relleno empleados en cada tipo de cemento ^23^^,^^24^. Además, el uso de un 3% de 4-META, seguido por un 5% de 10-MDP, mejoró significativamente la resistencia flexural ^16^^,^^25^. Sin embargo, las diferentes concentraciones de MDP tuvieron un impacto complicado sobre el módulo de elasticidad ^26^^,^^27^. Estos estudios indican que diversos tipos de cementos resinosos, que suelen variar en composición, exhiben valores distintos de resistencia flexural. Los resultados resaltan la importancia de continuar evaluando la resistencia de los diversos materiales de cementos resinosos disponibles en el mercado.

Investigaciones recientes han abordado la resistencia flexural de diversos tipos de cementos resinosos, y abarcan formulaciones comerciales, modificadas y patentadas. Ling *et al*. y Furuichi *et al*. ^28^ encontraron que un cemento resinoso con tecnología patentada superó en resistencia flexural a varios cementos comerciales. Por otro lado, estudios liderados por Skapska *et al*. ^26^ han investigado las propiedades mecánicas de un cemento resinoso autoadhesivo, lo cual revela que el uso de resina calentada representa una opción de cementación ventajosa en comparación con el cemento resinoso autoadhesivo RelyX U200.

La investigación en cementos resinosos autoadhesivos ha identificado diversos factores que afectan sus propiedades mecánicas, lo que influye en la elección del cemento según las necesidades clínicas y las condiciones de aplicación ^2^^,^^8^. Los odontólogos deben mantenerse al día con las investigaciones para tomar decisiones informadas, lo que resalta la importancia de seguir evaluando la resistencia flexural de los materiales disponibles ^29^. Por tanto, la presente investigación tuvo como objetivo evaluar la resistencia flexural de tres cementos resinosos dentales. La hipótesis nula a evaluar fue que no existe diferencia significativa en la entre los cementos resinosos: RelyX U200, Relyx^TM^ Universal y Duo-Link Universal.

## MATERIALES Y MÉTODOS

La investigación utilizó un método de observación estructurada y el diseño fue experimental *in vitro*. Fue aprobado por el comité de ética de la Universidad Científica del Sur (POS-115-2024-0220). Se prepararon veinte especímenes de los tres cementos resinosos dentales: RelyX U200 (3M/ESPE), Relyx^TM^ Universal (3M/ESPE) y Duo-Link Universal (Bisco Dental), los cuales se dividieron en tres grupos, de acuerdo a su marca (n = 20) (Tabla 1).

**Tabla 1:** Cementos resinosos utilizadas en esta investigación

Cemento	Matriz orgánica	Relleno	Color	Fabricante	Lote	Fecha de vencimiento
Relyx U200	Esteres de ácidos fosfóricos metacrilados, trietilenglicol dimetacrilato (TEGDMA), dimetacrilatos, p-toluensulfinato de sodio	Fibra de vidrio, sílice silano, persulfato de sodio, hidróxido de calcio	A1	3M ESPE, St. Paul, MN, EE. UU.	10815418	21/08/2025
Relyx^TM^ Universal	TEGDMA, uretano dimetacrilato (UDMA), hidróxidoperóxido, iniciador, pigmentos, hidroxietil metacrilato (HEMA). Sistema iniciador redox anfifílico (AIS)	Dióxido de titanio, sílice tratado con silano	A1	3M ESPE, St. Paul, MN, EE. UU.	10289331	03/09/2025
Duo-Link Universal	Base de bisfenol A-glicidil metacrilato (Bis-GMA), TEGDMA, trietilenglicol dimetacrilato, dimetacrilato de uretano y relleno vítreo, metacrilato tetrahidrofurfurio, Trimetacrilato Trimetilopropano 3-(trimetoxililo) propilo-2-metilo-2-ácido propendido	Fluoruro de iterbio, iterbio óxido-sílica	A1	BISCO, Inc. Irving Park Road Schaumburg. EE. UU.	2300111275	02/10/2025

Los especímenes fueron fabricados en matrices de 25 x 2 x 2 mm, siguiendo las instrucciones del fabricante y las especificaciones ANSI/ADA (ADA-CDMIE, 1993) ^30^. Los cementos fueron agregados dentro de un molde de acero inoxidable colocado entre dos platinas de vidrio para obtener el espesor uniforme. Estos especímenes fueron fotoactivados por 40 segundos con una lámpara LED Valo (Ultradent Products, Inc., South Jordan, UT, EE. UU.), con una radiación emitida de 1200 mW/cm^2^ y una punta de tamaño de 9,5 mm (+- 0,1 mm). Los excesos fueron eliminados y pulidos con discos Soflex (3M ESPE) con diferentes grados de abrasión y, finalmente, fueron almacenados en agua destilada a 37 °C por 24 horas. Las medidas finales de las barras de cemento fueron verificadas con un calibrador digital (Digimatic, Mitutoyo Corp., Tokio, Japón). 

La resistencia flexural se midió con la prueba de flexión de tres puntos, de acuerdo con la norma ISO 4049, en una máquina de ensayo universal (Instron®, Barcelona, España), a una velocidad de 0,5 mm/min hasta la fractura. La resistencia flexural (MPa) se calculó utilizando la siguiente fórmula: 

σ = 3*FL*

 2*bh* ^*2*^

Donde:

*F* es la carga aplicada.

*L* es la longitud de la barra en el tramo de apoyo de dos puntos.

*b* es el ancho de la sección transversal de la barra.

*h* es la altura de la sección transversal.

El análisis estadístico se realizó con el programa Python V3.20 (EE. UU.) (https://www.python.org). La distribución de datos se analizó usando la prueba de Shapiro-Wilk. La prueba de significancia estadística se hizo mediante Anova de un factor y comparaciones múltiples de Tukey p < 0,05.

## RESULTADOS

La resistencia flexural encontrada para el cemento RelyX U200 fue de 68,1±9,2 MPa. Para el cemento Relyx^TM^ Universal fue de 73,98±7,62 MPa y para el cemento Duo-Link Universal, de 104,7±7,8 MPa. Tras la comparación de los tres cementos se evidencia que existen diferencias significativas en el análisis de Anova de un factor con p < 0,001. Al realizar la prueba de comparaciones múltiples de Tukey, RelyX U200 y Duo-Link Universal mostraron diferencias estadísticamente significativas (p < 0,001), al igual que Relyx^TM^ Universal y Duo-Link Universal (p < 0,001). Entre Relyx^TM^ Universal y RelyX U200, no se encontró una diferencia estadísticamente significativa (Tabla 2).

**Tabla 2:** Evaluación comparativa de la resistencia flexural de los tres cementos: RelyX U200, RelixTM Universal y Duo-Link Universal

Grupo	Cemento	Resistencia flexural (MPa)	p1	p2
1	RelyX U200	68,1±9,2	<0.001	0,001 (entre grupo 1 y 3)
2	Relyx^TM^ Universal	73,98±7,62		0,079 (entre grupo 1 y 2)
3	Duo-Link Universal	104,7±7,8		0,001 (entre grupo 2 y 3)

p1: test Anova de un factor

p2: test de comparaciones múltiples de Tukey+

## DISCUSIÓN

Este estudio se enfoca en evaluar la resistencia flexural de tres cementos resinosos: RelyX U200, Relyx^TM^ universal y Duo-Link Universal. Se elaboraron barras de cemento que fueron sometidas a fuerzas externas en tres puntos, siguiendo la norma ISO 4049. Luego de examinar los resultados, el análisis estadístico determinó que la resistencia flexural de los tres cementos fueron estadísticamente diferentes. En promedio, la resistencia flexural del cemento Duo-Link Universal superó a los otros dos cementos. A pesar de que el cemento RelyX U200 es muy empleado en las cementaciones dentales, los hallazgos de esta investigación muestran que el cemento Duo-Link Universal es una alternativa confiable en la práctica dental.

La principal diferencia entre los tres cementos podría residir en la presencia, proporciones, matriz resinosa, cantidad de bis-GMA y naturaleza específica de los metacrilatos de uretodimetacrilato. Estudios previos han identificado estos componentes como clave para mejorar la adhesión y las propiedades mecánicas, incluyendo la resistencia flexural. Es probable que la mayor proporción de estos metacrilatos en Duo-Link Universal explique su superior resistencia flexural comparada con el cemento RelyX U200. Aunque tanto Duo-Link Universal como Relyx^TM^ Universal contienen metacrilatos de uretodimetacrilato, las diferencias en sus proporciones o naturaleza específica podrían influir en la formación de la red de polímero durante la fotoactivación, lo que afectaría la resistencia flexural ^31^.

De acuerdo con la norma ISO 4049, se indica un mínimo de resistencia flexural de 50 MPa para materiales de cementación. Por tanto, en el presente estudio se evidencia que todos los cementos evaluados cumplen por encima de dichas especificaciones. En el estudio de Carek *et al*. ^9^ se midió, entre otros parámetros, la resistencia flexural después de 28 días, y se reportaron similares resultados del desempeño para el cemento Relyx ^TM^ Universal.

Del mismo modo, en comparación con el estudio de Ramos *et al*. ^20^, que evaluó la resistencia flexural en el cemento RelyX U200 a diferentes concentraciones en el contenido de relleno inorgánico, en el que se evidencia una similitud resistencia flexural frente a lo obtenido en el presente estudio con el cemento RelyX U200. Estas similitudes se deben, posiblemente, a una modificación en el relleno inorgánico y se utilizó una polimerización dual que mejora las propiedades mecánicas y físicas.

En comparación con Rodrigues *et al*. ^23^ quienes evaluaron la influencia de la composición inorgánica y la morfología de las partículas de relleno en las propiedades mecánicas de los diferentes cementos resinosos, se evidenció que el cemento RelyX Unicem2, equivalente en nuestro estudio a RelyX U200, reporta una resistencia flexural ligeramente superior. Esto se debe a que estos cementos contienen partículas irregulares de diferentes tamaños, pequeñas y grandes, cuya combinación favorece una buena adaptación al llenar todos los espacios en la matriz de resina. Sin embargo, Furuichi *et al*. ^28^ determinaron las propiedades mecánicas y las características de resistencia al desgaste por impacto de cementos autoadhesivos. En este estudio se evaluó RelyX Unicem2, el cual mostró una resistencia flexural superior al RelyX U200. Esto se debería a que el contenido del relleno del cemento RelyX Unicem2 pasa por un proceso de calentamiento entre 25 y 800 °C, lo que podría influir en el aumento de sus propiedades mecánicas.

Los hallazgos de este estudio están limitados a la propiedad mecánica de resistencia flexural de tres cementos resinosos. En este aspecto, a pesar de que los resultados favorecen ampliamente al cemento Duo-Link Universal, estos podrían variar con referencia a las otras propiedades mecánicas (compresión, tracción, dureza, entre otras). En este sentido, se sugiere investigar las otras propiedades mecánicas de estos materiales para tener una mejor comprensión de ellos. Este estudio contribuye a la distinción de las características de resistencia flexural de tres cementos de uso común. Sin embargo, los resultados de este estudio se limitan al rango de parámetros y condiciones utilizados en este trabajo. Asimismo, frente a la creciente demanda de uso de cementos, futuras investigaciones deben explorar también otros tipos de resinas, métodos de curación y otras propiedades.

Es importante tener en cuenta estas limitaciones al interpretar los resultados de la investigación, y no generalizar los hallazgos. Sin embargo, al reconocer estas limitaciones, se puede tomar medidas para minimizar su impacto y mejorar la precisión de los resultados. Futuras investigaciones deberían analizar en detalle la composición de los cementos, enfocándose en las proporciones exactas de los componentes de la matriz resinosa y la naturaleza de las partículas de relleno, para comprender mejor su relación con la resistencia flexural.

## CONCLUSIÓN

El cemento resinoso que presenta mayor resistencia flexural es el Duo-Link Universal, en comparación con los cementos RelyX U200 y Relyx ^TM^ Universal. Mientras que el cemento RelyX U200 y Relyx ^TM^ Universal, no tienen diferencia estadísticamente significativa en cuanto a resistencia flexural.

## Financiamiento:

Autofinanciado
